# Aggression Amongst Outpatients With Schizophrenia and Related Psychoses in a Tertiary Mental Health Institution

**DOI:** 10.3389/fpsyt.2021.777388

**Published:** 2022-01-03

**Authors:** Anitha Jeyagurunathan, Jue Hua Lau, Edimansyah Abdin, Saleha Shafie, Sherilyn Chang, Ellaisha Samari, Laxman Cetty, Ker-Chiah Wei, Yee Ming Mok, Charmaine Tang, Swapna Verma, Siow Ann Chong, Mythily Subramaniam

**Affiliations:** ^1^Research Division, Institute of Mental Health, Singapore, Singapore; ^2^Department of Developmental Psychiatry, Institute of Mental Health, Singapore, Singapore; ^3^Department of Mood and Anxiety, Institute of Mental Health, Singapore, Singapore; ^4^Department of Psychosis, Institute of Mental Health, Singapore, Singapore

**Keywords:** aggression, BPAQ, schizophrenia, factor structure, confirmatory factor analysis

## Abstract

**Aims:** Aggression is defined as “any behavior intended to cause physical, emotional, or psychological harm to another.” The aims of the current study were to (i) examine underlying factor structure of the Buss-Perry Aggression Questionnaire (BPAQ) and (ii) explore socio-demographic and clinical correlates (symptom severity, substance use and alcohol use) among patients with schizophrenia and related psychoses in a multi-ethnic Asian population.

**Methods:** Data collected from 397 participants who were seeking outpatient treatment for schizophrenia and related psychoses at a tertiary psychiatric hospital were included in the analyses. BPAQ, a 29-item, four-factor instrument that measures physical aggression, verbal aggression, anger and hostility was used to assess aggression. Data on socio-demographic variables, age of onset of illness, drug use, alcohol use and symptom severity were also collected. Confirmatory factor analysis (CFA) was performed to establish the underlying factor structure of the BPAQ. Multiple regression analyses were utilized to examine socio-demographic and clinical correlates of the BPAQ factors.

**Results:** The mean age of the participants was 36.2 years (SD = 10.9, range: 21–65). Factor structure obtained from the CFA indicated that a higher order four-factor solution had an acceptable fit to the observed data (WLSMV χ^2^ = 1,025.35, df = 320, RMSEA = 0.07, CFI = 0.94, TLI = 0.93, SRMR = 0.05). Females had lower physical aggression and hostility scores as compared to males. Those with lower education had higher physical aggression scores as compared to those with higher education. Participants who received a diagnosis after the age of 30 years had higher physical aggression and anger scores as compared to those who received a diagnosis at or before 20 years of age. Symptom severity was positively associated with higher BPAQ scores.

**Conclusion:** The study findings demonstrated high internal consistency and applicable measurement factor structure of BPAQ in this study sample, making it an appropriate questionnaire for assessing aggressive behavior in this population. We also identified socio-demographic and clinical factors that were associated with aggression in patients with schizophrenia and related psychoses.

## Introduction

Mental illnesses are a leading cause of disability worldwide and about 25% of the world's population develop mental illness at some stage of their life (1). Various studies have shown an increased prevalence of aggressive behavior among persons with serious mental illness as compared to persons without such illness (2–4).

Schizophrenia is a chronic and severe mental illness affecting 20 million people worldwide (5). A recent study conducted in Singapore reported that ~2.3% of Singapore's general adult population are affected by schizophrenia and related psychoses in their lifetime (6). People with schizophrenia are commonly perceived as unpredictable and dangerous (7), with fears of being injured by an individual with severe mental illness being fairly common in the general public (8). However, such negative attitudes increase self-stigma among people with severe mental disorders leading to poor treatment outcomes (9, 10) perpetuating a cycle of stigma.

Individuals with schizophrenia have a higher rate of aggressive behavior (4 – 6 times higher) than the general population (11, 12). The association between aggressive behavior and mental illness is highly complex, more so when a patient with psychoses' inner world is imprecise and his or her belief or reality is affected (13). Prior studies have found that aggressive behavior is associated with clinical factors such as positive symptoms (14), failure to treat patients with schizophrenia (15), incomplete adherence to medication (16), as well as social factors such as lack of empathy and emotional detachment (17). Studies have also shown that substance-related disorders are significantly associated with aggressive behavior during a psychotic episode (18, 19). Nationwide psychosis studies from the United Kingdom and the Netherlands have reported that substance use disorders were closely related to increased rates of aggressive behavior (20). Research over the last few years has also reported high rates of victimization in people with severe mental health problems. People with mental disorders are more frequently victims of violence, rather than perpetrators of violence (21, 22). Victimized individuals are more likely to have worsening of symptoms, engage in alcohol or substance use to cope with the trauma, all of which may lead to violent behavior (21, 23).

Socio-demographic factors have been examined in relation to aggressive behavior among patients with schizophrenia. Some studies have reported that males scored significantly higher on the physical aggression, verbal aggression and hostility subscales (24, 25). A study conducted by Sher et al. (26) reported that participants with lower education showed more aggressive behavior (26). Similarly, others have concluded that younger age groups (27), widowed status and lower socioeconomic status (28) are associated with higher aggressive behavior among patients with schizophrenia.

While many researchers in Western countries have studied aggression in patients with schizophrenia and related psychoses, few have examined aggression among this group in Singapore. Singapore is a multi-ethnic, city-state located in Southeast Asia with a population of 4.04 million in 2020 (29). A 2005 study done in Singapore established that 37.0% of patients with first episode psychosis had aggression (13.7% had severe aggression and 23.3% had lesser aggression) (13). The authors found a significant association between aggression and duration of untreated illness. The multi-ethnic population, low prevalence of alcohol and other substance use disorders (30, 31), relatively low treatment gap (32) and high medication compliance (33), emphasize the need for local data in Singapore. Thus, the current study aims to (i) examine underlying factor structure of the Buss-Perry Aggression Questionnaire (BPAQ) and (ii) explore the socio-demographic and clinical associations (symptom severity, drug and alcohol use) of aggression as assessed by BPAQ among patients with schizophrenia and related psychoses in a multi-ethnic Asian population.

## Method

The present study used data from a cross-sectional survey that was conducted at the Institute of Mental Health (IMH) between October 2019 and March 2021. IMH is the sole tertiary care psychiatric hospital in Singapore that serves a patient population with a wide range of mental illnesses. The study commenced in October 2019 but was suspended during the lockdown period (April 2020–June 2020) in Singapore due to the Coronavirus pandemic. It was resumed in June 2020 while adhering to safe distancing and masking policies, while also providing participants with the option to participate via an online platform. The informed consent form and questionnaire were administered via the ZOOM platform if the online mode was preferred.

A total of 400 participants were recruited through convenience sampling. However, three cases were excluded due to: (i) recruiting the same person twice, (ii) one participant requested to withdraw from the study, and (iii) one participant was above the age limit of 65 years. Hence, a final sample size of 397 was utilized. Patients seeking treatment at IMH outpatient clinics and affiliated satellite clinics were enrolled in the study. The study included patients who were Singapore residents (including Singapore Citizens and Permanent Residents); aged 21–65 years; belonging to Chinese, Malay, Indian or Other ethnic groups; capable of providing consent; able to understand and read English; and having a clinical diagnosis of schizophrenia and related psychoses, as determined by a psychiatrist using Diagnostic and Statistical Manual of Mental Disorders, fourth edition (DSM-IV) criteria (34). Patients who had intellectual disabilities or cognitive impairment, or were not literate in English were excluded from the study. Study brochures informing attending patients of the ongoing study and the eligibility criteria were placed in the Clinician's rooms along with the phone numbers and email addresses of the study team members. Emails were sent to all treating clinicians and other health care professionals who were requested to refer eligible patients to participate in the study. All participants provided written informed consent prior to their participation. Data were collected through self-administered questionnaires which participants completed either on physical copies or online via a QuestionPro link (an option provided since June 2020 due to Coronavirus pandemic). Each participant took ~45–60 mins to complete the survey.

The study was initiated after receiving ethics approval from the relevant institutional ethics review board [National Healthcare Group Domain Specific Review Board (NHG DSRB Ref: 2019/00918)]. The research was carried out in accordance with the Declaration of Helsinki and the ethical principles of the Belmont Report.

## Measures

All participants completed a study questionnaire which included the following instruments:

1. *Socio-demographic questionnaire*: This included data on age, gender, ethnicity, marital status, education and monthly personal income. Clinical history was collected through a medical records review, which included information on age of onset of illness and clinical diagnosis of participants.
2. *Buss-Perry Aggression Questionnaire (BPAQ)* (25) is a 29-item, four-factor instrument that measures physical aggression, verbal aggression, anger, and hostility. Aggression was measured by the total score of the BPAQ and scores of the subscales including physical aggression (items 1–9), verbal aggression (items 10–14), anger (items 15–21) and hostility (items 22–29). The total BPAQ score could range from 29 to 145 with higher levels of aggression indicated by higher scores. Each question on the BPAQ employs a five-point Likert rating scale, where 1 = extremely uncharacteristic of me, 2 = somewhat uncharacteristic of me, 3 = neither uncharacteristic nor characteristic of me, 4 = somewhat characteristic of me, and 5 = extremely uncharacteristic of me. Subscale scores were derived by summing the ratings for the questions that define each of the subscales. The four-factor conceptualization of aggression is considered a gold standard for the measurement of aggression (35). The first two factors i.e., physical and verbal aggression represents the instrumental components. The third factor of anger implies physiological activation and represents the emotional component, the fourth factor of hostility involves feelings of opposition and injustice thus representing the cognitive component. The researchers suggest that anger acts as psychological bridge connecting instrumental and cognitive components (25).
3. *Symptoms Checklist- 90 Revised (SCL-90-R)* is a widely used 90-item psychiatric symptoms checklist that assesses psychiatric symptomatology measure of general psychiatric distress. It is a self-report instrument consisting of a series of 90 descriptions of symptoms that participants rate in terms of their severity (ranging from 0 = Not at all, to 4 = Extremely). The SCL-90-R is scored and interpreted in terms of nine primary symptom dimensions and three global indices of distress. A global severity index (GSI) can be obtained by taking the average of all 90-items, with higher scores reflecting higher distress and severity of symptoms. The primary symptom dimensions and global indices are labeled as follows: Somatization (SOM); Obsessive-Compulsive (OC); Interpersonal Sensitivity (IS); Depression (DEP); Anxiety (ANX); Hostility (HOS); Phobic Anxiety (PHOANX); Paranoid Ideation (PI); and Psychoticism (PSY). A large number of studies have been conducted demonstrating the reliability, validity and utility of the instrument (36). Psychometric evaluations have reported good internal consistency (alpha coefficients 0.77–0.90), test-retest reliability, and concurrent, construct, and discriminant validity (37). It is one of the most widely used measures of psychological distress in clinical practice and research. The internal consistency reliability for SCL-90-R (Cronbach's alpha = 0.99) was high in the current study.
4. *Cut-annoyed-guilty-eye (CAGE) questionnaire* (38): The CAGE questionnaire uses four items to assess self-reported problems related to alcohol use in the present sample. The 4 items were prefaced by a screening question, “Was there ever a period in your life when you drank at least 12 drinks in a year?” (a drink was defined as “a glass of wine, a can/bottle of beer, or a shot/jigger of liquor either alone or in a mixed drink”). Participant who indicated they had never had an alcoholic drink or drank less than 12 drinks per year were directed to skip the CAGE items. Those who answered “yes” to the screening question were asked about 4 aspects of their drinking habits: (1) Feeling that they should *cut down* on their drinking; (2) Being *annoyed* about criticism of their drinking; (3) Feeling bad or *guilty* about their drinking; and (4) Having a drink first thing in the morning to steady their nerves or to get rid of a hangover (*eye opener*). Endorsing two or more items in the CAGE tool was indicative of problematic alcohol use in this study. The questionnaire has demonstrated high test-retest reliability and adequate convergent validity (39) and has been validated in Singapore among older adults (40). A moderate internal consistency of this scale was obtained for this sample (Cronbach's alpha = 0.69).
5. *Drug Abuse Screen Test (DAST-10)* (41) is a brief screening instrument that can be used in clinical and non-clinical populations to detect potential drug abuse, which excludes alcohol or tobacco use, during the past 12-months. This 10-item tool may be self-administered or interviewer-administered. 1 point is scored for each “yes” response, except for question 3 to which a “no” response receives 1 point. Scores of 0–2 indicate low-level or no drug use problems; 3–5 indicates moderate problems; 6–8 indicates substantial problems and 9–10 severe problems. In the current study, a cut-off of ≥3 was applied to indicate problematic drug use (42). Internal consistency reliability of DAST-10 (Cronbach's alpha = 0.73) was high in the current study.

### Statistical Analysis

Analyses in the present study were conducted with MPlus version 8.2 and Stata version 15. Mean and standard deviation are presented for continuous variables while frequencies and percentages are displayed for categorical variables. Confirmatory factor analysis (CFA) and unidimensional exploratory factor analysis (EFA) were estimated and tested using MPlus to evaluate the factor structure of the BPAQ. As the items of the BPAQ were measured on an ordinal scale, a weighted-least-square with mean- and variance-adjusted (WLSMV option) estimation was used to model the polychoric correlation matrix (CATEGORICAL option). The following fit indices were utilized to compare the overall fit of the CFA models and their complexities: (i) root mean square error of approximation (RMSEA), (ii) comparative fit index (CFI), (iii) Tucker-Lewis index (TLI), (iv) Standardized Root Mean Square Residual (SRMR). Both CFI and TLI values range from 0 to 1, with higher values representing better fit. CFI values above 0.95 and TLI values above 0.90 are considered to be of excellent fit (43). With regard to RMSEA, values below 0.08 indicate moderate fit, while values of 0.05 or less indicate close fit to the observed data (44). Standardized root mean squared residual (SRMR) values were also evaluated, which indicates acceptable fit when values are smaller than 0.08 (43, 44). The unidimensional EFA employed a WLSMV estimator and an oblique (GEOMIN) rotation. Internal consistency of measures was assessed *via* Cronbach's alpha values. First order and second order CFA models were tested. Items of the BPAQ were summed to form a total score and their four respective factor scores based upon results of the final CFA model. Five multiple regression analyses were conducted using the “enter” method to examine correlates of the BPAQ total score and its individual factors. Correlates entered into these regression models include socio-demographic variables (i.e., age, gender, ethnicity, education, marital status, and age of onset) and clinical variables (i.e., problematic drug use, alcohol use, and SCL-90-R global severity index). Following this, five multiple regression analyses were run in which the nine subscales of the SCL-90-R (i.e., somatization, obsessive-compulsive, interpersonal sensitivity, depression, anxiety, hostility, phobic-anxiety, paranoid ideation, and psychoticism) were entered into each model while adjusting for the effects of socio-demographic and clinical variables to further examine the association between symptom severity and aggression factors.

## Results

### Socio-Demographic and Clinical Characteristics of the Sample

Socio-demographic and clinical characteristics of the sample are presented in Table 1. The mean age of participants (*n* = 397) was 36.2 ± 10.9 years, (range: 21–65), with the majority being Chinese (*n* = 297, 74.8%), single (*n* = 320, 80.6%), with highest education level of A-level/Polytechnic/Vocational School/Institute of Technical Education (ITE) (*n* = 182, 45.8%), and with monthly personal income of below 2,000 (in Singapore dollars) (*n* = 202, 50.9%). Approximately half of the sample were males (*n* = 201, 50.6%).

**Table 1:** Socio-demographic and clinical characteristics of sample (*n* = 397).

	***N***	**%**
**Age groups**		
21–34	198	49.9
35–49	146	36.8
50–65	53	13.4
**Gender**		
Male	201	50.6
Female	196	49.4
**Ethnicity**		
Chinese	297	74.8
Malay	51	12.9
Indian	38	9.6
Others	11	2.8
**Marital status**		
Single	320	80.6
Married	47	11.8
Separated/divorced/widowed	30	7.6
**Education level**		
Primary and below	18	4.5
Secondary	120	30.2
A-level/Polytechnic/Vocational school/ITE^*^	182	45.8
Degree and above	77	19.4
**Monthly personal income in Singapore dollars**		
No Income	114	28.7
Below 2,000	202	50.9
2,000-3,999	48	12.1
4,000 and above	15	3.8
Refused/Don't know^*^	18	4.5
**Age of onset of diagnosis**		
20 years and younger	108	27.2
21–25	114	28.7
26–30	79	19.9
31 and older	96	24.2
**Drug abuse screen test (DAST-10)**		
No problematic drug use	371	93.5
Has problematic drug use	23	5.8
Refused/Don't know^*^	3	0.8
**Cut-annoyed-guilty-eye (CAGE) questionnaire**		
No lifetime drinking problems	376	94.7
Has lifetime drinking problems	21	5.3
	**Mean**	**SD**
**Buss-Perry aggression questionnaire (BPAQ)**		
BPAQ anger	14.34	5.5
BPAQ physical aggression	19.68	7.6
BPAQ verbal aggression	9.89	3.7
BPAQ hostility	21.63	7.5
BPAQ total	65.46	21.6
**Symptoms check list-90-revised (SCL)**		
SCL_Global Severity Index	0.94	0.9
SCL_somatization	0.74	0.7
SCL_obsessive compulsive	1.25	1.0
SCL_interpersonal sensitivity	1.08	1.0
SCL_depression	1.14	1.0
SCL_anxiety	0.895	1.0
SCL_hostility	0.64	0.8
SCL_phobic anxiety	0.696	1.0
SCL_paranoid ideation	6.29	6.4
SCL_psychoticism	0.87	1.0

* Refused/don't know responses were considered as missing data. ^*^ITE, Institute of Technical Education.

The mean age of onset of diagnosis of illness was 26.1 years (SD = 8.1), 5.8% had problematic drug use (*n* = 23), and 5.3% had problematic lifetime drinking (*n* = 21). Mean BPAQ total score was 65.45, (SD = 21.6). Mean SCL-90-R Global severity index score was 0.94 (SD = 0.9).

### Confirmatory Factor Analysis in the Overall Sample

Descriptive information regarding the 29 items of the BPAQ can be found in Supplementary Table 1. A CFA was first conducted utilizing the model suggested by its developers (25), in which a four-factor model with 29 items was tested. However, upon examination of the CFA model, and a unidimensional EFA model, two items were removed. Firstly, item 9 “I am an even-tempered person” was removed from subsequent iterations for the following reasons: (i) it demonstrated a poor standardized factor loading (0.053) with its respective factor within the initial first order CFA model, (ii) examination of the underlying polychoric correlation matrix revealed that this item had poor correlations (ranging from −0.121 to 0.290) with all other items, and (iii) the unidimensional EFA with all 29 items indicated that item 9 had a factor loading of 0.06, below a recommended cut-off of 0.4 (45), indicating that the item performs poorly in the unidimensional assessment of aggression. Secondly, item 4 “I tell my friends openly when I disagree with them” was also removed as it had a factor loading of 0.36 within the unidimensional EFA. Estimates of the 29-item unidimensional EFA can be found in Supplementary Table 2. A CFA of a first-order four-factor model using the remaining 27-items indicated a solution with acceptable fit (WLSMV χ^2^ = 1027.41, df = 318, RMSEA = 0.075, CFI = 0.94, TLI = 0.93, SRMR = 0.05). In order to support the generation of a total score, a single higher order four-factor model was also tested. Standardized factor loadings of the single higher order four factor solution and model's corresponding fit statistics can be found in Table 2. Results indicate that the higher order four-factor solution had acceptable fit to the observed data (WLSMV χ^2^ = 1,025.35, df = 320, RMSEA = 0.07, CFI = 0.94, TLI = 0.93, SRMR = 0.05) and was thus chosen as the final solution. No modification indices were specified. Standardized factor loadings of the items to their respective factors were moderate to high, ranging from 0.47 to 0.84. All four factors loaded strongly with the higher order factor, with values ranging from 0.89 to 0.97. In the calculation of the factor scores, the present study opted to sum without any reverse scoring. Internal consistency assessed by Cronbach alpha values was high: Total score (27-items; α = 0.94), Anger (6 items; α = 0.84), Physical Aggression (9 items; α = 0.85), Verbal Aggression (4-items; α = 0.76), Hostility (8 items; α = 0.86). It is to note that although item 16 “I can think of no good reason for ever hitting a person” was negatively worded, it demonstrated a positive and acceptable standardized factor loading (0.47) with its respective factor of Physical Aggression”, and was thus summed in the calculation of that factor.

**Table 2:** Standardized factor loadings and fit indices of the higher order four factor model.

**Item number**	**Item description and their corresponding factors**	**Standardized factor loadings**
	**Anger** (6 items; α = 0.84; Higher order factor loading: 0.968)	
1	Some of my friends think I am a hot head (a person who is easily angered)	0.657
12	I flare up quickly but get over it quickly	0.663
18	I have trouble controlling my temper	0.814
19	When frustrated, I let my irritation show	0.708
23	I sometimes feel like a powder keg (barrel of gunpowder) ready to explode	0.841
28	Other people always seem to get the breaks (favorable opportunities)	0.801
	**Physical aggression** (9 items; α = 0.85; Higher order factor loading: 0.912)	
2	If I have to resort to violence to protect my rights, I will	0.657
5	I have become so mad that I have broken things	0.678
8	Once in a while, I can't control the urge to strike another person	0.812
11	I have threatened people I know	0.732
13	Given enough provocation (intentional teasing or torment), I may hit another person	0.774
16	I can think of no good reason for ever hitting a person	0.469
22	If somebody hits me, I hit back	0.701
25	There are people who pushed me so far that we came to blows (physical fights or a serious argument)	0.795
29	I get into fights a little more than the average person	0.809
	**Verbal Aggression** (4 items; α = 0.76; Higher order factor loading: 0.968)	
6	I can't help getting into arguments when people disagree with me	0.734
14	When people annoy me, I may tell them what I think of them	0.570
21	I often find myself disagreeing with people	0.785
27	My friends say that I'm somewhat argumentative	0.778
	**Hostility** (8 items; α = 0.86; Higher order factor loading: 0.889)	
3	When people are especially nice to me, I wonder what they want	0.724
7	I wonder why sometimes I feel so bitter about things	0.712
10	I am suspicious of overly friendly strangers	0.627
15	I am sometimes eaten up with jealousy	0.722
17	At times I feel I have gotten a raw deal (unfair treatment) out of life	0.737
20	I sometimes feel that people are laughing at me behind my back	0.771
24	Other people always seem to get the breaks (favorable opportunities)	0.753
26	I know that “friends” talk about me behind my back	0.744
**Goodness of fit indices of CFA models**	**First order four factor model**	**Higher order four factor model**
Chi-square test of Model fit	χ^2^ (318) = 1,027.41, *p* <0.001	χ^2^ (320) = 1,025.35, *p* <0.001
RMSEA	0.075	0.074
CFI	0.939	0.939
TLI	0.933	0.934
SRMR	0.051	0.052

*All standardized factor loadings were significant at p < 0.001*.

### Socio-Demographic and Clinical Correlates of Aggression

Results of the multiple regression analyses can be found in Table 3. Females had lower scores of physical aggression (β = −1.6; *p* = 0.04) and hostility (β = −1.6; *p* = 0.03) compared to male participants with schizophrenia. In terms of education, those who had lower education levels (i.e., primary and below, secondary, A-level/polytechnic/vocational school/ITE) had higher scores in physical aggression (β = 4.6; *p* = 0.03, β = 2.4; *p* = 0.04, β = 2.1; *p* = 0.05 respectively) when compared to those with a qualification of an undergraduate degree and above. Participants who had an age of onset of diagnosis after the age of 30 years (31 years or older) had higher total BPAQ (β = 7.4; *p* = 0.03) and physical aggression scores (β = 2.7; *p* = 0.03) when compared to those whose age of onset was 20 years and below. Additionally, those whose age of onset was 21–25 years (β = 1.8; *p* = 0.02) and 31 years and older (β = 2.6; *p* = 0.003), had higher anger scores. Higher scores on the SCL-90-R Global Symptoms Index were positively associated with higher sub domain scores and total score of BPAQ (*p* < 0.001).

**Table 3:** Socio-demographic and clinical correlates of Buss-Perry Aggression Questionnaire (BPAQ).

**Variables**	**Buss-Perry aggression questionnaire**								
	**Total**			**Physical aggression**			**Verbal aggression**		
	β^*^	**95%CI**	***p***	β^*^	**95%CI**	***p***	β^*^	**95%CI**	***p***
**Age groups**									
21–34	Reference			Reference			Reference		
35–49	1.1	(−3.9 to 6.1)	0.66	0	(−1.8 to 1.8)	1	0.5	(−0.4 to 1.4)	0.27
50–65	0.6	(−6.6 to 7.7)	0.88	−0.2	(−2.8 to 2.4)	0.88	0.6	(−0.6 to 1.8)	0.35
**Gender**									
Male	Reference			Reference			Reference		
Female	−3.2	(−7.5 to 1.0)	0.14	−1.6	(−3.1 to −0.0)	**0.04**	−0.4	(−1.1 to 0.4)	0.35
**Ethnicity**									
Chinese	Reference			Reference			Reference		
Malay	−2.1	(−8.7 to 4.5)	0.53	−0.3	(−2.8 to 2.1)	0.79	0.1	(−1.0 to 1.3)	0.81
Indian	−1.9	(−9.3 to 5.4)	0.6	0.6	(−1.9 to 3.3)	0.63	−0.4	(−1.7 to 0.8)	0.52
Others	1.6	(−11.5 to 14.7)	0.81	0.9	(−3.9 to 5.7)	0.72	−0.4	(−2.7 to 1.9)	0.71
**Marital status**									
Single	Reference			Reference			Reference		
Married	−0.2	(−6.5 to 6.5)	0.99	−1.1	(−3.5 to 1.3)	0.39	0.8	(−3.1 to 2.0)	0.15
Separated/Divorced/Widowed	2.9	(−5.5 to 11.3)	0.5	−0.2	(−3.2 to 2.9)	0.9	1.2	(−0.3 to 2.6)	0.11
**Education level**									
Degree and above	Reference			Reference			Reference		
Primary and below	7.9	(−4.0 to 19.7)	0.19	4.6	(0.4 to 8.8)	**0.03**	0.8	(−1.2 to 2.8)	0.45
Secondary	2.8	(−3.6 to 9.2)	0.39	2.4	(0.1 to 4.8)	**0.04**	−0.01	(−1.1 to 1.1)	0.98
A-level/Polytechnic/ Vocational school/ITE^*^	4.1	(−1.6 to 9.9)	0.16	2.1	(0.0 to 4.2)	**0.05**	0.4	(−0.6 to 1.4)	0.41
**Monthly personal income (in singapore dollars)**									
Below 2,000	Reference						Reference		
2,000–3,000	−2.3	(−8.7 to 4.1)	0.47	−0.9	(−3.3 to 1.4)	0.44	−0.2	(−1.3 to 0.9)	0.75
4,000 and above	−5.4	(−16.3 to 5.5)	0.33	−2.8	(−6.8 to 1.2)	0.17	−0.7	(−2.6 to 1.2)	0.46
No income	−5.8	(−5.3 to 4.2)	0.81	−0.8	(−2.5 to 0.9)	0.37	0.02	(−0.8 to 0.8)	0.96
**Age of onset of diagnosis**									
20 years and younger	Reference			Reference			Reference		
21–25	1.5	(−4.2 to 7.1)	0.61	0.1	(−1.9 to 2.2)	0.903	−0.1	(−1.1 to 0.9)	0.88
26–30	2.2	(−4.0 to 8.4)	0.48	0.8	(−1.4 to 3.1)	0.472	0.03	(−1.0 to 1.1)	0.95
31 and older	7.4	(0.8 to 14.0)	**0.03**	2.7	(0.3 to 5.1)	**0.029**	0.3	(−0.9 to 1.4)	0.64
**Cut-annoyed-guilty-eye (CAGE) questionnaire**									
No drinking problems	Reference			Reference			Reference		
Lifetime drinking problems	7.2	(−2.4 to 16.8)	0.14	2.6	(−0.9 to 6.1)	0.14	1	(−1.1 to 2.1)	0.32
**Drug abuse screen test (DAST−10)**									
No problematic drug use	Reference			Reference			Reference		
Has problematic drug use	1.8	(−7.7 to 11.3)	0.71	1	(−2.5 to 4.3)	0.58	1	(−0.6 to 2.7)	0.23
**Global severity index (GSI)**	10.9	(8.4 to 13.4)	**<0.001**	2.8	(1.9 to 3.7)	**<0.001**	1.6	(1.2 to 2.0)	**<0.001**
**Variables**	**Buss-Perry aggression questionnaire**					
	**Anger**			**Hostility**		
	β^*^	**95%CI**	***p***	β^*^	**95%CI**	***p***
**Age groups**						
21–34	Reference			Reference		
35–49	−0.3	(−1.6 to 1.0)	0.68	0.7	(−1.0 to 2.4)	0.41
50–65	0	(−1.9 to 1.8)	0.96	0.4	(−2.1 to 2.8)	0.76
**Gender**						
Male	Reference			Reference		
Female	0.4	(−0.7 to 1.5)	0.47	−1.6	(−3.0 to −0.1)	**0.03**
**Ethnicity**						
Chinese	Reference			Reference		
Malay	−0.7	(−2.4 to 1.1)	0.45	−1.2	(−3.5 to 1.0)	0.29
Indian	−1.6	(−3.5 to 0.3)	0.09	−0.2	(−2.6 to 2.3)	0.9
Others	0.2	(−3.2 to 3.6)	0.91	1.1	(−3.4 to 5.5)	0.64
**Marital status**						
Single	Reference			Reference		
Married	1	(−0.7 to 2.7)	0.25	−0.7	(−3.0 to 1.5)	0.51
Separated/divorced/widowed	0.4	(−1.8 to 2.5)	0.73	2.1	(−0.7 to 4.9)	0.14
**Education level**						
Degree and above	Reference			Reference		
Primary and below	2.9	(−0.1 to 5.9)	0.06	0.6	(−3.5 to 4.6)	0.77
Secondary	1.5	(−0.2 to 3.2)	0.08	−1.3	(−3.5 to 0.9)	0.25
A-level/Polytechnic/Vocational school/ITE^*^	1	(−0.5 to 2.5)	0.18	0.5	(−1.4 to 2.5)	0.59
**Monthly personal income (in Singapore dollars)**						
Below 2,000	Reference			Reference		
2,000–3,000	−0.8	(−2.5 to 0.9)	0.35	−0.4	(−2.6 to 1.8)	0.72
4,000 and above	−2	(−4.9 to 0.8)	0.16	0.2	(−3.6 to 3.9)	0.93
No income	−0.3	(−1.5 to 1.0)	0.66	0.4	(−1.2 to 2.0)	0.64
**Age of onset of diagnosis**						
20 years and younger	Reference			Reference		
21–25	1.8	(0.4 to 3.3)	**0.02**	−0.3	(−2.2 to 1.7)	0.79
26–30	1.4	(−0.2 to 3.0)	0.085	−0.2	(−2.3 to 1.9)	0.85
31 and older	2.6	(0.9 to 4.3)	**0.003**	1.8	(−0.4 to 4.1)	0.11
**Cut-annoyed-guilty-eye (CAGE) questionnaire**						
No drinking problems	Reference			Reference		
Lifetime drinking problems	1.1	(−1.4 to 3.6)	0.37	2.8	(−0.5 to 6.1)	0.09
**Drug abuse screen test (DAST-10)**						
No problematic drug use	Reference			Reference		
Has problematic drug use	1.1	(−1.3 to 3.5)	0.38	−1	(−4.2 to 2.1)	0.52
**Global severity index (GSI)**	2.6	(2.0 to 3.3)	**<0.001**	3.8	(3.0 to 4.7)	**<0.001**

* ITE, Institute of Technical Education; ^*^Multiple regression analyses. Results in bold indicate significant findings (p < 0.05).

### Symptom Severity (SCL-90-R) and Aggression (BPAQ)

Results of the multiple regression analyses examining the nine subscales of symptoms severity (i.e., SCL-90-R) and their association with aggression, after adjusting for other socio-demographic and clinical correlates are presented in Table 4. OC subscale of SCL-90-R was positively associated with the anger domain (β = 1.8; *p* = 0.003). Higher IS scores were associated with higher scores in the BPAQ domains of verbal aggression (β = 1.3; *p* = 0.01) and hostility (β = 2.7; *p* = 0.003). The HOS subscale was positively associated with the total (β = 10.7; *p* < 0.001), physical aggression (β = 4.8; *p* < 0.001), verbal aggression (β = 1.3; *p* = 0.002) and anger domains (β = 3.6; *p* < 0.0001) of the BPAQ. Higher PI subscale scores were associated with higher scores in the hostility (β = 0.5; *p* < 0.0001) domain and BPAQ total (β = 0.7; *p* = 0.049). In contrast, PHOANX was negatively associated with BPAQ total (β = −4.6; *p* = 0.03), anger (β = −1.2; *p* = 0.03) and hostility (β = −1.7; *p* < 0.02) domains.

**Table 4:** Symptoms severity (SCL-90-R) and Buss-Perry Aggression Questionnaire (BPAQ).

**Symptoms checklist (SCL)**	**Buss-Perry aggression questionnaire**														
	**Total**			**Physical aggression**			**Verbal aggression**			**Anger**			**Hostility**		
	**β**	**95%CI**	***p***	**β**	**95%CI**	***p***	**β**	**95%CI**	***p***	**β**	**95%CI**	***p***	**β**	**95%CI**	***p***
SCL_somatization	−2.7	(−7.6 to 2.2)	0.28	−1.1	(−2.9 to 0.7)	0.23	0.5	(−0.4 to 1.3)	0.29	−0.6	(−1.8 to 0.7)	0.37	−1.0	(−2.6 to 0.6)	0.23
SCL_obsessive compulsive	3.8	(−0.7 to 8.3)	0.1	0.9	(−0.7 to 2.6)	0.27	0.4	(−0.4 to 1.2)	0.28	1.8	(0.6 to 2.9)	**0.003**	0.6	(−0.9 to 2.1)	0.42
SCL_interpersonal sensitivity	4.3	(−1.0 to 9.6)	0.11	−0.6	(−2.5 to 1.4)	0.58	1.3	(−0.4 to 2.3)	**0.01**	0.8	(−0.6 to 2.2)	0.24	2.7	(0.9 to 4.4)	**0.003**
SCL_depression	−3.1	(−8.4 to 2.2)	0.25	−1.2	(−3.1 to 0.8)	0.23	−0.8	(−1.7 to 0.2)	0.1	−0.8	(−2.2 to 0.6)	0.24	−0.3	(−2.1 to 1.5)	0.75
SCL_anxiety	−0.8	(−6.7 to 5.2)	0.79	0.4	(−1.8 to 2.6)	0.75	−0.6	(−1.7 to 0.4)	0.25	−0.9	(−2.4 to 0.7)	0.28	0.5	(−1.5 to 2.5)	0.63
SCL_hostility	10.7	(5.9 to 15.5)	**<0.001**	4.8	(3.0 to 6.6)	**<0.001**	1.3	(0.5 to 2.2)	**0.002**	3.6	(2.3 to 4.8)	**<0.0001**	0.7	(−0.9 to 2.3)	0.39
SCL_phobic anxiety	−4.6	(−8.9 to −0.4)	**0.03**	−0.9	(−2.5 to 0.6)	0.25	−0.7	(−1.4 to 0.03)	0.06	−1.2	(−2.3 to −0.1)	**0.03**	−1.7	(−3.1 to −0.3)	**0.02**
SCL_paranoid ideation	0.7	(0.002 to 1.4)	**0.049**	0.2	(−0.1 to 0.5)	0.13	0.1	(−0.04 to 0.2)	0.16	−0.1	(−0.2 to 0.1)	0.58	0.5	(0.2 to 0.7)	**<0.0001**
SCL_psychoticism	−0.4	(−5.5 to 4.6)	0.86	−0.1	(−2.0 to 1.7)	0.89	−0.2	(−1.1 to 0.6)	0.62	0.7	(−0.6 to 1.9)	0.31	−1	(−2.7 to 0.7)	0.25

*Results of the multiple regression analyses examining the nine subscales of symptoms severity and their association with aggression, after adjusting for other sociodemographic and clinical correlates. Results in bold indicate significant findings (p < 0.05)*.

## Discussion

To our knowledge the current study is the first that has established the factor structure and correlates of BPAQ in a multi-ethnic Asian sample of patients with schizophrenia and related psychoses. Our findings indicate that the factor structure of the BPAQ scale in the overall sample was somewhat similar to that proposed by the developers (25), with some differences in the verbal aggression and anger subscales. This study also identified important socio-demographic and clinical correlates of the BPAQ scale among those with schizophrenia. These findings are discussed in further detail in the subsequent sections.

### Factor Structure and Internal Consistency of the BPAQ

The results suggest that the total and subscale scores of BPAQ had an adequate reliability in terms of internal consistency, and provide a valid measurement of physical aggression, verbal aggression, anger and hostility in the sample. A number of researchers have investigated the reliability and validity of this instrument, and the findings generally point in the direction of good psychometric properties with the qualification that the elimination of a few items improves the instrument; Harris et al. (46) removed two items from the hostility subscale, Gerevich et al. (35) removed two reversed scored items, Zimonyi et al. (47) removed item 21 and item 27, while Nakano (48) removed three items. To develop a more appropriate measurement model, the present study identified a 27-item, four-factor measurement model with acceptable goodness-of-fit and high internal consistency, despite the removal of two items (item 9 from anger subscale and item 4 from the verbal aggression subscale). The current study findings further suggest the removal of these items for cross-cultural use, as the aforementioned studies have also suggested excluding items from the BPAQ.

### Correlates of BPAQ Among Patients With Schizophrenia and Related Psychoses

Females were significantly associated with lower aggressive scores in physical and hostility subscales of BPAQ compared to male patients with schizophrenia and related psychoses. The findings were similar to various other research studies (25, 49–51). A possible explanation could be the strong relation of testosterone with aggression (49). The literature reports more physical aggression in men than women in the general population (52, 53), as well as among patients with mental disorders (25, 54).

Research studies have shown that individuals with lower education show greater aggressive behavior among patients with severe mental disorders (26, 28, 55). Our findings were similar to other studies where those with primary education displayed more physical aggression (28, 55, 56). A possible explanation provided by Harris and Kelly (56) was that higher education might serve to reduce aggression, as it teaches individuals to consider alternative points of view, to inhibit immediate emotional responses in favor of thoughtful ones, and to plan for long-term goals. In addition, individuals who have lower aggression and are more willing to accept gratification may be more likely to seek higher education (56). It is also possible that lower education might represent an indirect proxy of worse cognitive functioning; cognitive impairment in turn has been reported to be associated with aggressive and violent behavior among patients with schizophrenia (57, 58). This raises an interesting possibility as cognitive impairment could benefit from cognitive remediation therapy (59). Cognitive training and social cognitive training have been shown to be effective in reducing violence in patients with schizophrenia, which is encouraging (60) although further research is needed.

The current study found that a later onset of illness (31 years and above) was significantly associated with higher BPAQ total scores, physical aggression and anger scores among patients with schizophrenia and related psychoses. To our knowledge, this study is among the first to report a positive association between the aggression scores and older age of onset of schizophrenia. A possible explanation could be the association of aggression with a variety of negative outcomes in older adults, including social isolation, unemployment, and low socioeconomic status (61, 62). Our current study collected data on age of onset based on the date of diagnosis of patients, however some patients may experience symptoms much before receiving the actual diagnosis and this may contribute to a longer duration of untreated illness. Hodgins et al. (63) suggested that substance misuse, increase in dopamine production that leads to stress dysregulation, and changes in brain structure as a result of the prolonged illness may increase the risk of physical aggression towards others during the course of illness. It is plausible that older patients in our study may have had a later onset of illness and the illness was associated with adverse outcomes that increased the risk of aggression in these patients. Future studies should include information on social support and functional outcomes to better understand the relationship between age of onset and aggressive behavior.

Various studies have suggested an association between alcohol, drug use and aggressive behavior among psychiatric and general population (64, 65). In contrast, substance use was not significantly associated with aggression in the current study. A possible explanation could be that the strict measures toward misuse of drugs in Singapore, with tough penalties and capital punishment associated with it, resulted in low substance use or under-reporting of substance use in the present study due to fear or social desirability bias; thus, resulting in a lack of significant association with aggression. A similar finding of low prevalence of substance use was also seen in prior studies conducted among clinical population (30, 31).

SCL-90-R Global Severity Index score was positively correlated with the total and all four subscales scores of BPAQ. Our results found self-reported symptoms severity, more specially, obsessive compulsive, hostility, and paranoid ideation symptom dimensions were significantly associated with higher aggression scores. This finding is consistent with many studies suggesting that positive symptoms of schizophrenia, more specifically paranoid ideations, and hostility are commonly the link between schizophrenia and aggressive acts (66–69). The potential explanations for the association between paranoid ideation and aggression are well established whereby individuals with paranoid symptoms have an attentional bias for threat cues (70), a tendency to jump to conclusions (68) and attribute hostile intent to others' actions. Individual with paranoid symptoms may utilize aggression as a safety behavior in an effort to maintain their “safety” or prevent threats (67).

### Limitations

Limitations of the current study include the recruitment of all patients from a single tertiary psychiatric hospital who may not be representative of patients with schizophrenia in general. Secondly, the participants in this study were recruited using a convenience sampling method and were restricted to only those who were self-referred or referred by treating clinicians or other health care professionals; hence, they might not be representative of all outpatients in the institution and that could impose a sampling bias. Thirdly, the cross-sectional design cannot demonstrate causal relations between variables. Fourth, the data about actual aggressive behaviors were not captured at all; future studies might want to look at hospital reports of actual violence. Lastly, as noted earlier, although item 16 was a negatively worded item, it loaded positively with the factor of physical aggression. It may be that the double negative within the question (i.e., no good reason) may have confused respondents and caused this positive factor loading. Future studies utilizing the BPAQ should aim to rework this question by removing the negatively worded phrasing, since the remaining 27 items in the final model put forth in the present study are all worded positively.

## Conclusion

In conclusion, our research established the factor structure of the BPAQ in an Asian population among outpatients with schizophrenia and related psychoses seeking treatment at a tertiary hospital. The study findings demonstrate high internal consistency and a valid factor structure of BPAQ, making it appropriate for assessing aggression in this population. We identified socio-demographic and clinical factors that were associated with aggression in patients with schizophrenia and related psychoses. Early implementation of risk protocols as well as psycho-education in this vulnerable group may potentially reduce the risk of aggression in patients with psychosis. Our study results highlight the relevance of adapting BPAQ instrument in the hospital setting to enable implementation of targeted interventions and holistic programmes for patients. However, larger community based studies are needed to replicate our findings.

## Data Availability Statement

Data may be available upon reasonable request and subjected to approval by the Institutional Review Board (IRB). This is a requirement mandated for this research study by our IRB and funders. Requests to access the dataset should be directed to the senior author, Mythily Subramaniam, mythily@imh.com.sg.

## Ethics Statement

The studies involving human participants were reviewed and approved by National Healthcare Group Domain Specific Review Board. The patients/participants provided their written informed consent to participate in this study.

## Author Contributions

AJ wrote the manuscript, conceived the study, and wrote the protocol. JHL analyzed the data, assisted in the interpretation of the findings, and provided comments for the article. EA reviewed the analyses and provided comments for the article. SS, SC, ES, LC, K-CW, YMM, CT, and SV critically reviewed the article. SAC and MS reviewed the protocol and critically reviewed the article. All authors provided intellectual input and approved the final manuscript.

## Funding

The study was funded through the Singapore Ministry of Health's National Medical Research Council under the Centre Grant Programme (Ref No.: NMRC/CG/M002/2017_IMH). The funding body had no role in the design of the study and data collection, analysis, and interpretation of data and in writing the manuscript.

## Conflict of Interest

The authors declare that the research was conducted in the absence of any commercial or financial relationships that could be construed as a potential conflict of interest.

## Publisher's Note

All claims expressed in this article are solely those of the authors and do not necessarily represent those of their affiliated organizations, or those of the publisher, the editors and the reviewers. Any product that may be evaluated in this article, or claim that may be made by its manufacturer, is not guaranteed or endorsed by the publisher.
