# Supplementary material for: Aggression Amongst Outpatients With Schizophrenia and Related Psychoses in a Tertiary Mental Health Institution
Source: Front Psychiatry. 2022 Jan 3;12:777388. doi: 10.3389/fpsyt.2021.777388 (PMC8761620; doi:10.3389/fpsyt.2021.777388)
Supplement: Supplementary file 1 [file Data_Sheet_1.docx]

**Supplementary Table 1**

| **Descriptive statistics of the Buss-Perry Aggression Questionnaire (BPAQ)** | | | | | | | | | | |
| --- | --- | --- | --- | --- | --- | --- | --- | --- | --- | --- |
| **Items** | **Extremely Uncharacteristic** | | **Somewhat Uncharacteristic** | | **Neither Uncharacteristic Nor Characteristic** | | **Somewhat Characteristic** | | **Extremely Characteristic** | |
|  | N | % | N | % | N | % | N | % | N | % |
| 1. Some of my friends think I am a hot head (a person who is easily angered) | 124 | 31.2 | 96 | 24.2 | 104 | 26.2 | 57 | 14.4 | 15 | 3.8 |
| 2. If I have to resort to violence to protect my rights, I will | 142 | 35.8 | 74 | 18.6 | 88 | 22.2 | 66 | 16.6 | 27 | 6.8 |
| 3. When people are especially nice to me, I wonder what they want | 83 | 20.9 | 79 | 19.9 | 115 | 29.0 | 92 | 23.2 | 28 | 7.1 |
| 4. I tell my friends openly when I disagree with them* | 42 | 10.6 | 64 | 16.1 | 117 | 29.5 | 125 | 31.5 | 49 | 12.3 |
| 5. I have become so mad that I have broken things | 167 | 42.1 | 72 | 18.1 | 76 | 19.1 | 56 | 14.1 | 25 | 6.3 |
| 6. I can’t help getting into arguments when people disagree with me | 113 | 28.5 | 113 | 28.5 | 93 | 23.4 | 57 | 14.4 | 21 | 5.3 |
| 7. I wonder why sometimes I feel so bitter about things | 98 | 24.7 | 75 | 18.9 | 89 | 22.4 | 100 | 25.2 | 34 | 8.6 |
| 8. Once in a while, I can’t control the urge to strike another person | 213 | 53.7 | 69 | 17.4 | 73 | 18.4 | 30 | 7.6 | 12 | 3.0 |
| 9. I am an even-tempered (not easily annoyed or made angry) person. ( R ) * | 43 | 10.8 | 78 | 19.7 | 92 | 23.2 | 124 | 31.2 | 60 | 15.1 |
| 10. I am suspicious of overly friendly strangers | 83 | 20.9 | 64 | 16.1 | 110 | 27.7 | 94 | 23.7 | 43 | 10.8 |
| 11. I have threatened people I know | 197 | 49.6 | 75 | 18.9 | 75 | 18.9 | 35 | 8.8 | 15 | 3.8 |
| 12. I flare up quickly but get over it quickly | 97 | 24.4 | 79 | 19.9 | 97 | 24.4 | 83 | 20.9 | 41 | 10.3 |
| 13. Given enough provocation (intentional teasing or torment), I may hit another person | 175 | 44.1 | 77 | 19.4 | 83 | 20.9 | 43 | 10.8 | 19 | 4.8 |
| 14. When people annoy me, I may tell them what I think of them | 83 | 20.9 | 89 | 22.4 | 104 | 26.2 | 89 | 22.4 | 32 | 8.1 |
| 15. I am sometimes eaten up with jealousy | 113 | 28.5 | 82 | 20.7 | 102 | 25.7 | 70 | 17.6 | 29 | 7.3 |
| 16. I can think of no good reason for ever hitting a person. ( R ) | 145 | 36.5 | 66 | 16.6 | 84 | 21.2 | 54 | 13.6 | 48 | 12.1 |
| 17. At times I feel I have gotten a raw deal (unfair treatment) out of life | 88 | 22.2 | 60 | 15.1 | 100 | 25.2 | 95 | 23.9 | 54 | 13.6 |
| 18. I have trouble controlling my temper | 136 | 34.3 | 101 | 25.4 | 95 | 23.9 | 46 | 11.6 | 19 | 4.8 |
| 19. When frustrated, I let my irritation show | 83 | 20.9 | 80 | 20.2 | 104 | 26.2 | 100 | 25.2 | 30 | 7.6 |
| 20. I sometimes feel that people are laughing at me behind my back | 114 | 28.7 | 82 | 20.7 | 85 | 21.4 | 79 | 19.9 | 37 | 9.3 |
| 21. I often find myself disagreeing with people | 107 | 27.0 | 98 | 24.7 | 115 | 29.0 | 53 | 13.4 | 23 | 5.8 |
| 22. If somebody hits me, I hit back | 123 | 31.0 | 74 | 18.6 | 103 | 25.9 | 61 | 15.4 | 36 | 9.1 |
| 23. I sometimes feel like a powder keg (barrel of gunpowder) ready to explode | 170 | 42.8 | 78 | 19.7 | 88 | 22.2 | 39 | 9.8 | 22 | 5.5 |
| 24. Other people always seem to get the breaks (favourable opportunities) | 102 | 25.7 | 68 | 17.1 | 121 | 20.5 | 68 | 17.1 | 38 | 9.6 |
| 25. There are people who pushed me so far that we came to blows (physical fights or a serious argument) | 173 | 43.6 | 73 | 18.4 | 73 | 18.4 | 50 | 12.6 | 28 | 7.1 |
| 26. I know that "friends" talk about me behind my back | 120 | 30.2 | 76 | 19.1 | 94 | 23.7 | 69 | 17.4 | 38 | 9.6 |
| 27. My friends say that I’m somewhat argumentative | 139 | 35.0 | 86 | 21.7 | 105 | 26.4 | 50 | 12.6 | 17 | 4.3 |
| 28. Sometimes I fly off the handle (to suddenly become very angry) for no good reason | 187 | 47.1 | 71 | 17.9 | 82 | 20.7 | 40 | 10.1 | 17 | 4.3 |
| 29. I get into fights a little more than the average person | 220 | 55.4 | 70 | 17.6 | 82 | 20.7 | 15 | 3.8 | 10 | 2.5 |
| (R): Items that were reverse coded  * two Items were removed from the final CFA model | | | | | | | | | | |

**Supplementary Table 2**

| **Results of a unidimensional Exploratory Factor Analysis** | | |
| --- | --- | --- |
| **Item number** | **Item description** | **Factor Loading** |
| 1 | Some of my friends think I am a hot head (a person who is easily angered) | 0.63 |
| 2 | If I have to resort to violence to protect my rights, I will | 0.63 |
| 3 | When people are especially nice to me, I wonder what they want | 0.68 |
| 4 | I tell my friends openly when I disagree with them* | 0.36 |
| 5 | I have become so mad that I have broken things | 0.64 |
| 6 | I can’t help getting into arguments when people disagree with me | 0.70 |
| 7 | I wonder why sometimes I feel so bitter about things | 0.67 |
| 8 | Once in a while, I can’t control the urge to strike another person | 0.77 |
| 9 | I am an even-tempered (not easily annoyed or made angry) person* | 0.06 |
| 10 | I am suspicious of overly friendly strangers | 0.59 |
| 11 | I have threatened people I know | 0.70 |
| 12 | I flare up quickly but get over it quickly | 0.64 |
| 13 | Given enough provocation (intentional teasing or torment), I may hit another person | 0.74 |
| 14 | When people annoy me, I may tell them what I think of them | 0.56 |
| 15 | I am sometimes eaten up with jealousy | 0.68 |
| 16 | I can think of no good reason for ever hitting a person | 0.45 |
| 17 | At times I feel I have gotten a raw deal (unfair treatment) out of life | 0.69 |
| 18 | I have trouble controlling my temper | 0.78 |
| 19 | When frustrated, I let my irritation show | 0.68 |
| 20 | I sometimes feel that people are laughing at me behind my back | 0.72 |
| 21 | I often find myself disagreeing with people | 0.75 |
| 22 | If somebody hits me, I hit back | 0.67 |
| 23 | I sometimes feel like a powder keg (barrel of gunpowder) ready to explode | 0.81 |
| 24 | Other people always seem to get the breaks (favourable opportunities) | 0.71 |
| 25 | There are people who pushed me so far that we came to blows (physical fights or a serious argument) | 0.76 |
| 26 | I know that "friends" talk about me behind my back | 0.70 |
| 27 | My friends say that I’m somewhat argumentative | 0.74 |
| 28 | Other people always seem to get the breaks (favourable opportunities) | 0.77 |
| 29 | I get into fights a little more than the average person | 0.77 |
| * indicates two Items that were removed due to having loadings below cut-off of 0.4 | | |
